# Analgesic Effects of Intraperitoneal Bupivacaine Following Laparoscopic Bariatric Surgery: A Randomized Clinical Trial

**DOI:** 10.1007/s11695-026-08818-8

**Published:** 2026-07-01

**Authors:** Sherif-Abd AlSayed Abd AlRahman Taie, Amir K. Abosayed, Ahmed-Yahia Abd ElDayem, Amr Mohamed AbdelFattah Ayad

**Affiliations:** Surgery Department, Faculty of Medicine, Cairo University, Cairo, Egypt

**Keywords:** Bariatric surgery, Obesity, Intraperitoneal instillation, Postoperative pain

## Abstract

**Background:**

Postoperative pain is a significant concern following laparoscopic bariatric surgery. Effective analgesia is essential to facilitate recovery, reduce complications, and minimize opioid consumption, especially in patients with obesity who are more susceptible to respiratory compromise. This randomized clinical trial aimed to evaluate the efficacy of weight-standardized intraperitoneal instillation of local anesthetic (IPILA) using 0.2% bupivacaine in reducing postoperative pain among patients undergoing laparoscopic bariatric procedures.

**Methods:**

Ninety adult patients undergoing bariatric surgery were randomly assigned to either an intervention group receiving intraperitoneal bupivacaine (0.5 mL/kg) or a control group receiving no instillation. Pain scores were measured using the Visual Analogue Scale (VAS) at recovery, and 2, 4, and 24 h postoperatively. Secondary outcomes included analgesic and antiemetic use after surgery.

**Results:**

The intervention group showed significantly lower VAS scores at recovery (2.2 ± 0.6 vs. 4.5 ± 1.2) and at 2 h (2.7 ± 1.2 vs. 4.1 ± 1.1) compared to the control group (*p* < 0.001). No significant difference was observed at 4 and 24 h. Analgesic and antiemetic requirements were similar between groups.

**Conclusion:**

Intraperitoneal instillation of bupivacaine significantly reduces early postoperative pain after bariatric surgery without increasing complications or analgesic needs. While its effect does not extend beyond the immediate postoperative period, IPILA remains a simple, safe, and effective adjunct to multimodal analgesia protocols in bariatric patients for early postoperative pain control.

## Introduction

Obesity is one of the fastest-growing health problems that has reached epidemic proportions worldwide, with projections that over half of the global population may be overweight or obese by 2030 [1]. Metabolic and bariatric surgeries (MBS) are widely recognized as the most effective treatment for severe obesity and its related comorbidities. In the United States, the annual volume of bariatric operations increased from roughly 158,000 in 2011 to over 270,000 by 2023, reflecting the growing acceptance of laparoscopic bariatric surgery as a key therapy for obesity [2, 3].

However, postoperative pain represents a major challenge in clinical practice especially after bariatric surgeries since poorly managed pain can impair deep breathing and coughing, hinder early mobilization, and thereby increase the risk of pulmonary complications and thromboembolic events in this high-risk population. Moreover, patients with obesity frequently have obstructive sleep apnea (OSA), which makes them particularly vulnerable to opioid-induced respiratory depression [4, 5].

The national perioperative guidelines accordingly emphasize minimizing opioid use and employing multimodal analgesia in obese surgical patients to optimize pain relief, promote early mobilization, and reduce opioid-related respiratory complications [6]. Recently, intraperitoneal instillation of local anesthetic (IPILA) has emerged as a promising technique to enhance analgesia in laparoscopic surgery. This simple approach involves spraying a local anesthetic solution such as bupivacaine or ropivacaine into the peritoneal cavity at the end of the operation with gentle irrigation, to bathe visceral surfaces and block nociceptive afferents from the peritoneum. IPILA aims to reduce postoperative visceral pain and referred shoulder pain from pneumoperitoneum, thereby complementing other analgesic modalities [7, 8].

The aim of this study was to evaluate the efficacy of weight-standardized IPILA in patients undergoing laparoscopic bariatric surgery and determine its effects on post-operative pain outcomes.

## Patients and Methods

This randomized clinical trial included ninety adult patients with severe obesity aged 18 years or older with American Society of Anesthesiology (ASA) physical status grade I & II who underwent laparoscopic bariatric procedures including sleeve gastrectomy, one anastomosis gastric bypass (OAGB), Roux-en-Y gastric bypass (RYGB), single anastomosis duodenal-ileal bypass (SADI), and revision surgery in at our Hospital, between October 2022 and March 2023. Patients were excluded if they had an allergy to local anesthetic, severe heart disease like congestive heart failure, creatinine clearance less than 60 mL/h, or moderate to severe liver dysfunction classified as Child–Pugh score B or C. After approval from the Research and Ethics Committee, written informed consent was obtained from all participants before starting this study.

### Study Interventions and Procedures

#### Surgery

All patients underwent their procedure with the same surgeon and anesthetist at a single institution. Laparoscopic procedures were typically carried out using a 10-mm optical entry camera port, two 12–15-mm operating ports, a 5-mm Nathanson liver retractor port, and a 5-mm assistant port. Carbon dioxide insufflation was set to a pressure of 14 mmHg. At the end of procedure, a mixing cannula was used to apply a solution either normal saline or 0.2% bupivacaine directly over the oesophageal hiatus and in both subdiaphragmatic spaces. The drain was maintained clamped during the first hour after the instillation. The dose of 0.5 mL/kg of 0.2% bupivacaine (1 mg/kg total dose) was selected based on established safety profiles in patients with obesity, remaining below the recommended maximum dose of 2–3 mg/kg for bupivacaine to avoid local anesthetic systemic toxicity.

#### Anesthetic Protocol

Eligible patients underwent balanced general anesthesia. Standard monitoring was applied, including a 3-lead ECG, non-invasive blood pressure (NIBP), pulse oximetry, and neuromuscular function assessment. Invasive arterial monitoring was reserved for cases where NIBP readings were unreliable or inaccurate. Patients were positioned in a reverse Trendelenburg posture while anesthesia induction was performed using fentanyl at a dose of 2 mcg/kg, and tracheal intubation was facilitated with 0.5 mg/kg of atracurium following loss of consciousness. Anesthesia maintenance was carried out using 1–1.2% isoflurane in a mixture of oxygen and air, with atracurium administered at 0.1 mg/kg every 20 min.

At the start of surgery, patients received 8 mg of dexamethasone unless contraindicated due to diabetes and the use of oral hypoglycemics or insulin. At the end of the procedure, parecoxib (40 mg), droperidol (0.625 mg), and ondansetron (4 mg) were administered. Upon arrival to the post-anesthesia care unit (PACU), 2 g of paracetamol was given, with additional antiemetics and opioids (oxycodone or fentanyl in case of allergies) provided as needed. Postoperatively, patients were monitored in a ward setting with continuous oxygen saturation monitoring.

#### Study Outcomes

The Visual Analogue Scale (VAS). is a pain rating system that ranges from 0 (no pain) to 10 (worst severe pain).that can help patient care providers in determining the degree of pain following surgery.Assessing pain were blinded to group allocation by the nursing staff supervised by member of the research team by using VAS in the PACU immediately after operation, as well as at 2 and 4 h after surgery. Secondary endpoints evaluations were conducted at 6, 12, and 24 h postoperatively and continued until the patient was discharged. Additionally, the use of postoperative analgesics and antiemetics, and postoperative complications were recorded.

Routine pain management involved administering paracetamol 1 gram every 8 h. In cases of breakthrough pain, an additional 1 gram of paracetamol was given. A failed block was considered if the patient reported a VAS score greater than 4 or if the regular paracetamol regimen failed to control the pain. For patients who did not respond to the initial pain management strategy, the treatment plan included administering an intravenous non-steroidal anti-inflammatory drug (NSAID), such as ketorolac. If the pain persisted despite this, a diluted solution of pethidine (50 mg in 10 mL of saline) was administered, with 4 mL of this solution delivered to manage the pain effectively.

### Statistical Analysis

Data was analyzed using SPSS version 28. The normality of the data was tested using the Kolmogorov-Smirnov single-sample test. Qualitative data were expressed as counts and percentages. Associations between qualitative variables were analyzed using the Chi-square test. Quantitative data were reported as either mean with standard deviation (SD) or median with range, depending on the distribution. To compare between groups for quantitative variables, the Mann-Whitney test was used. A (*P* ≤ 0.05) was considered significant.

## Results

A total of 90 patients were enrolled, with a mean age of 35.4 ± 9.7 years (range: 19–60 years). The majority of participants were female, comprising 78.7% of patients. Additionally, 23.3% of patients had a history of diabetes, and 20% were hypertensive. The mean height was 165.1 ± 7.7 cm, preoperative weight 134.5 ± 19.9 kg, and BMI 48.8 ± 7.2. The majority of the participants (81.1%) underwent Sleeve gastrectomy (40 patients in intervention group and 33 in control group). There were no statistically significant differences between the intervention and control groups in terms of demographic or anthropometric data (Table 1).

**Table 1:** Demographic and clinical characteristics of the participants

Characteristics	Total (*n* = 90)	Control group (*n* = 45)	Intervention group (*n* = 45)	*p*-value
Age (years)				
Mean ± SD	35.4 ± 9.7	35.7 ± 9.4	35.1 ± 10.1	0.591
Median (range)	36.5 (19–60)	35 (19–60)	37 (20–60)	
Gender				
Male	19 (21.3%)	9 (20.0%)	11 (24.4%)	0.612
Female	70 (78.7%)	36 (80%)	34 (75.6%)	
Height (cm)				
Mean ± SD	165.1 ± 7.7	166 ± 8	165 ± 8	0.127
Median (range)	165 (150–183)	165 (151–183)	164 (150–180)	
Preoperative weight (Kg)				
Mean ± SD	134 ± 19.1	133.1 ± 20.7	134.5 ± 19.9	0.743
Median (range)	130 (100–195)	135 (90–180)	131 (90–195)	
Preoperative BMI (Kg/M2)				
Mean ± SD	48.8 ± 7.2	48.7 ± 7.5	49 ± 6.9	0.475 0.475
Median (range)	48.1 (34.6–68.4)	47.6 (36.7–68.4)	49.6 (34.6–64.4)	
Diabetes				
No	69 (76.7%)	34 (75.6%)	35 (79.5%)	0.803
Yes	21 (23.3%)	11 (24.4%)	10 (22.2%)	
Hypertension				
No	72 (80%)	37 (82.2%)	35 (77.8%)	0.598
Yes	18 (20%)	8 (17.8%)	10 (22.2%)	

### Postoperative Pain Outcomes

**Fig. 1:**
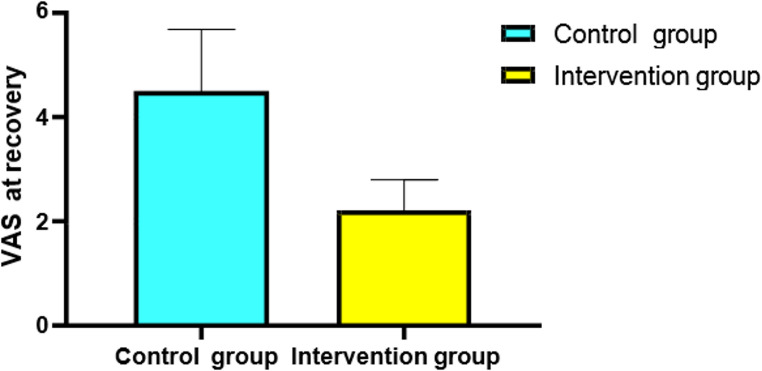
Comparison of VAS score at recovery between groups

**Fig. 2:**
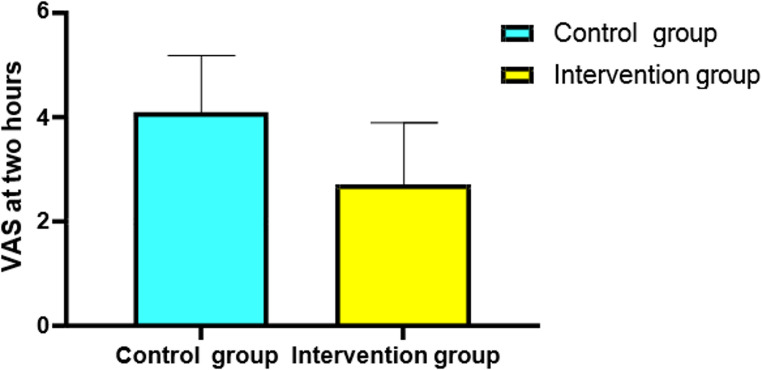
Comparison of VAS score between groups after 2 h

There was a significantly lower average VAS at recovery in the intervention compared to the control group (2.2±0.6 versus 4.5 ± 1.2, *p*<0.001) (Fig. 1). In addition, there was a significantly lower average VAS at two hours in the intervention compared to the control group (2.7 ± 1.2versus 4.1 ± 1.1, *p*<0.001). (Fig. 2). On the other hand, there was no significant difference between control and intervention groups regarding VAS- 4 h and VAS- 24 h after surgery (*p* = 0.845, and 0.838 respectively). Table 2.

**Table 2:** Visual analogue scale (VAS) for IPILA and control groups post-bariatric surgery

Characteristics	Total (*n* = 90)	Control group (*n* = 45)	Intervention group (*n* = 45)	*p*-value
VAS at recovery				
Mean ± SD	3.3 ± 1.4	4.5 ± 1.2	2.2±0.6	< 0.001
Median (range)	3.3 (2–6)	4 (2–6)	2 (2–4)	
VAS- 2 h				
Mean ± SD	3.4 ± 1.2	4.1 ± 1.1	2.7 ± 1.2	< 0.001
Median (range)	4 (2–6)	4 (2–6)	2 (2–4)	
VAS- 4 h				
Mean ± SD	3.1 ± 1.1	3.1 ± 1.2	3.1 ± 1.1	0.845
Median (range)	4 (2–6)	4 (2–6)	4 (2–4)	
VAS- 24 h				
Mean ± SD	2.4 ± 1.1	2.1 ± 1.1	2.7 ± 1.1	0.838
Median (range)	2 (0–4)	2 (0–4)	2 (0–4)	

### Need for postoperative analgesia and antiemetics

The majority of participants (91.1%) required the use of NSAIDs and/or opioids for postoperative pain management. Among those patients, approximately two-thirds (62.2%) needed more than one type of analgesic to achieve adequate pain control. Additionally, more than two-thirds of the participants required antiemetic medications to manage postoperative nausea and vomiting.

When comparing the control group with the intervention group, there were no statistically significant differences in the overall need for NSAIDs/opioids, the number of analgesic medications required, or the use of antiemetics (p-values were 0.459, 0.609, and 1.000, respectively), indicating comparable outcomes between both groups (Table 3).

**Table 3:** The need for postoperative medications between study groups

Characteristics	Total (*n* = 90)	Control group (*n* = 45)	Intervention group (*n* = 45)	*p*-value
Need NSAID/Opioids				
No	8 (8.9%)	3 (6.7%)	5 (11.1%)	0.459
Yes	82 (91.1%)	42 (93.3%)	40 (88.9%)	
Number of NSAID/Opioids (*n* = 82)				
One	31 (37.8%)	17 (40.5%)	14 (35%)	0.609
Two	51 (62.2%)	25 (59.5%)	26 (65%)	
Need of antiemetic				
No	30 (33.3%)	15 (33.3%)	15 (33.3%)	1.000
Yes	60 (66.7%)	30 (66.7%)	30 (66.7%)	

## Discussion

Pain following laparoscopic surgery is often linked to surgical procedures such as the introduction of carbon dioxide (CO₂) into the abdominal cavity. This gas insufflation stretches the peritoneum, irritates the diaphragm, and may leave residual gas after the operation, all of which can stimulate peritoneal nerves and lead to visceral and shoulder pain. Administering local anesthetics directly into the abdominal cavity helps block these visceral nerve signals, and the anesthetic’s absorption into the bloodstream may also contribute to lowering visceral pain perception [9, 10].

The findings of this clinical trial demonstrated a significant reduction in immediate postoperative pain scores at recovery phase immediately after surgery and at 2 h postoperatively in the intraperitoneal anesthesia group compared to the control group. However, this analgesic benefit did not extend beyond the early postoperative period with non-significant difference between 2 groups after 24 h. These results are consistent with previous studies that examined the role of IPILA in laparoscopic procedures. For example, Gupta et al. reported reduced early postoperative pain when bupivacaine was instilled in the peritoneal cavity after laparoscopic cholecystectomy [11]. Similarly, Omar et al. found that intraperitoneal local anesthetics reduced visceral pain after bariatric surgery compared to patients who did not receive it.

By directly targeting visceral pain pathways, IPILA aims to reduce postoperative visceral pain and referred shoulder pain from pneumoperitoneum, thereby complementing other analgesic modalities. The technique is simple, adds minimal operative time, and has shown a favorable safety profile. Recent studies in bariatric patients have reported that adding intraperitoneal local anesthetic does not increase postoperative complications, with no differences observed in rates of morbidity or mortality compared to control [12, 13].

Several studies on bariatric procedures including sleeve gastrectomy, RYGB, SADI, and gastric banding have evaluated intraperitoneal instillation of local anesthetics. Most show significant reductions in early postoperative pain, aligning with current study findings [14–16]. A study by Ruiz-Tovar et al. reported that intraperitoneal ropivacaine led to lower pain scores, reduced morphine use, earlier mobilization, and shorter hospital stay in sleeve gastrectomy and RYGB patients [17]. Moreover, Elsayed et al. found that combining intraperitoneal with surgical site bupivacaine injection extended the time to first analgesia request [18]. However, Schipper et al. reported no significant pain or opioid reduction with this technique, highlighting some variability in results [15].

Despite significant VAS score reduction early on, the study did not observe a statistically significant difference in analgesic consumption or antiemetic use between the groups. This suggests that while IPILA may provide an initial pain relief advantage, it does not obviate the need for systemic analgesia in the longer postoperative period. However, variations in surgical type, anesthetic concentration, and pain management protocols may explain this discrepancy.

This study has some limitations which are worthy of mention. Insufflation rate and pressure are key factors in postoperative pain, with studies like Ozdemir et al. showing that lower flow rates and pressures can reduce pain scores [19]; however, the current study used higher values (10 L/min at 14 mmHg) to maintain adequate surgical visibility, despite potential trade-offs. Moreover, Preoperative chronic pain and analgesic use did not significantly influence postoperative pain scores in this analysis, based on multivariable assessments. Additionally, pain perception is inherently subjective and varies between individuals, making standardized pain evaluation challenging.

## Conclusion

This randomized clinical trial demonstrates that intraperitoneal instillation of 0.2% bupivacaine significantly reduces immediate postoperative pain in patients undergoing laparoscopic bariatric surgery. Patients who received weight-adjusted bupivacaine reported lower VAS scores at recovery and at 2 h postoperatively, highlighting the effectiveness of IPILA as a simple and safe adjunct to multimodal analgesia. However, the analgesic benefit did not extend beyond the early postoperative period, with no significant differences in pain scores at 4 and 24 h, or in the overall use of analgesics or antiemetics between the groups. These findings suggest that while IPILA does not replace systemic analgesics, it can enhance early pain control and potentially improve patient comfort during the critical recovery phase.

### Stregnth and Limitations

The prospective randomised controlled design of this study strengthens it.However, the study is limited by the relatively small sample size.Further large-scale, multicenter studies are recommended to optimize dosing protocols and evaluate its integration with other pain management strategies for enhanced postoperative outcomes in bariatric patients.No adverse events related to local anesthetic systemic toxicity were observed, which may be attributable to the relatively low dose used and the exclusion of patients with impaired metabolic function.

## Data Availability

Data can be obtained upon request.
